# Early Results of Three-Year Monitoring of Red Wood Ants’ Behavioral Changes and Their Possible Correlation with Earthquake Events

**DOI:** 10.3390/ani3010063

**Published:** 2013-02-04

**Authors:** Gabriele Berberich, Martin Berberich, Arne Grumpe, Christian Wöhler, Ulrich Schreiber

**Affiliations:** 1 Department of Geology, Faculty of Biology, University Duisburg-Essen, 45141 Essen, Universitätsstr. 5, Germany; E-Mail: ulrich.schreiber@uni-due.de; 2 Am Plexer 7, 50374 Erftstadt, Germany; E-Mail: mb@berberichweb.com; 3 Image Analysis Group, Faculty of Electrical Engineering and Information Technology, TU Dortmund, Otto-Hahn-Straße 4, 44227 Dortmund, Germany; E-Mails: arne.grumpe@tu-dortmund.de (A.G.); christian.woehler@tu-dortmund.de (C.W.)

**Keywords:** red wood ants’ behavioral changes, earthquakes, long-term *in situ* monitoring, automated image evaluation routine, statistical analyses, tectonically active faults

## Abstract

**Simple Summary:**

For three years (2009–2012), two red wood ant mounds (*Formica rufa*-group), located at the seismically active Neuwied Basin (Eifel, Germany), have been monitored 24/7 by high-resolution cameras. Early results show that ants have a well-identifiable standard daily routine. Correlation with local seismic events suggests changes in the ants’ behavior hours before the earthquake: the nocturnal rest phase and daily activity are suppressed, and standard daily routine does not resume until the next day. At present, an automated image evaluation routine is being applied to the video streams. Based on this automated approach, a statistical analysis of the ant behavior will be carried out.

**Abstract:**

Short-term earthquake predictions with an advance warning of several hours or days are currently not possible due to both incomplete understanding of the complex tectonic processes and inadequate observations. Abnormal animal behaviors before earthquakes have been reported previously, but create problems in monitoring and reliability. The situation is different with red wood ants (RWA; *Formica rufa*-group (Hymenoptera: Formicidae)). They have stationary mounds on tectonically active, gas-bearing fault systems. These faults may be potential earthquake areas. For three years (2009–2012), two red wood ant mounds (*Formica rufa*-group), located at the seismically active Neuwied Basin (Eifel, Germany), have been monitored 24/7 by high-resolution cameras with both a color and an infrared sensor. Early results show that ants have a well-identifiable standard daily routine. Correlation with local seismic events suggests changes in the ants’ behavior hours before the earthquake: the nocturnal rest phase and daily activity are suppressed, and standard daily routine does not resume until the next day. At present, an automated image evaluation routine is being applied to the more than 45,000 hours of video streams. Based on this automated approach, a statistical analysis of the ants’ behavior will be carried out. In addition, other parameters (climate, geotectonic and biological), which may influence behavior, will be included in the analysis.

## 1. Introduction

One of the major challenges of seismology is to predict earthquakes. However, despite applying modern space and/or ground based technologies, a reliable method for short-term earthquake forecasting with reliable warnings several hours or days in advance is currently not possible and remains limited to only a few minutes before the quake happens. During the past decade, earthquake precursors, like ionospheric perturbations, electromagnetic phenomena, ground heating and composition changes of soil-efflux (radon, CO_2_, *etc.*), have been scientifically investigated. However, they are far from being successfully applicable, because these precursors are highly regionally variable depending not only on the large-scale tectonic main stress regime, but also on the small-scale tectonic regime [1,2,3]. 

Abnormal animal behaviors have been reported previously, but create problems in monitoring and reliability [4,5,6,7]. However, long-term *in situ* data collection, statistically significant validation of these precursors in focus regions over a long period of time and plausible scenarios explaining the evolution of such behaviors are still missing. 

**Figure 1:**
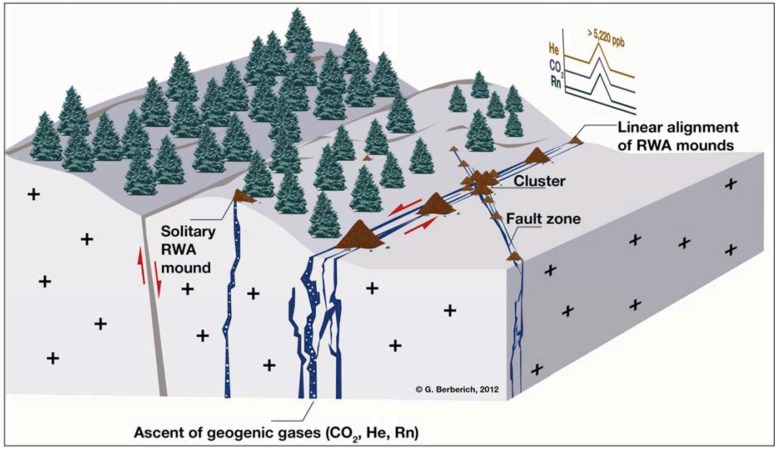
GeoBioScience: relation of red wood ant (RWA) mound sites and gas-permeable tectonic fault systems. Along the open systems, geogenic gas concentrations exceed atmospheric standards (He > 5,220 ppb) and background values (CO_2_ > 2 Vol%; Rn up to 110 Bq/L). Linear alignment of RWA mounds occurs if the gas-permeable fault is running open for a very long distance, e.g., 3 km at the Oberehe site (West Eifel, *cf.* Figure 2(a)). Here, mound numbers of 120 mounds in a line can be mapped. At intersections of two or more tectonic faults, clusters of RWA mounds occur. Solitary mounds can be found mostly at unusual locations with spotty degassing [16].

The situation is different with red wood ants (RWA; *Formica rufa*-group (Hymenoptera: *Formicidae*). A particular advantage of RWA is their high sensitivity to environmental changes. Forced by some million years of evolutionary selection, they may have developed anticipatory mechanisms [8,9]. Besides an extremely strong temperature sensitivity (0.25 K) [10], they have chemo-receptors for the detection of CO_2_-concentrations [11,12] and an electromagnetic field sensitivity [13,14,15]. Recent research in the West Eifel (West Germany), combined with geostatistical analyses, have demonstrated the correlation of soil gas anomalies, especially helium anomalies, as outstanding fault zone tracers and spatial distribution of red wood ant (RWA) mounds along tectonically active, gas-permeable faults (*cf.* Figure 1) [16,17].

**Figure 2:**
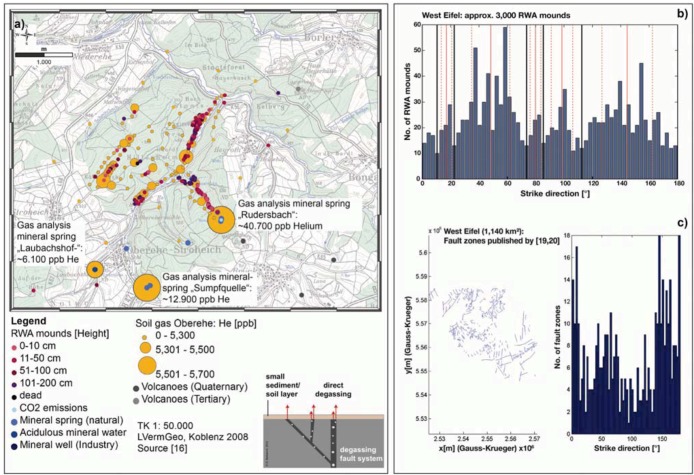
Relationship between helium anomalies (He > 5,220 ppb), fault zones and RWA mounds for the Oberehe site in the West Eifel (approx. 40 km distance from Koblenz, East Eifel); (**a**) showing helium anomalies (orange dots), RWA mounds (red dots), mineral springs (blue dots) and volcanoes (black dots); (**b**) showing alignment of the RWA mounds (degrees from N) and (**c**) fault zone directions [19,20](degrees from N). The comparison of both the spatial RWA mound distribution with the significantly increased gas anomalies confirms that the RWA colony is situated in a tectonically sheared region. Although the reference site is surrounded by various forest stands, no further RWA mounds were found in those forest stands during the area-wide mapping. These results suggest that RWA mounds can be used as biological indicators of active, gas-permeable faults [16,17].

At the reference location Oberehe (*cf.* Figure 2(a)), a site with small soil/sediment covers, a direct degassing of geogenic gases into the atmosphere occurs. The linear alignment of the RWA mounds directly corresponds to that of the helium anomalies. The Hough transform (a well-established algorithm for identifying linear structures in sets of points [18]) applied on the spatial distribution of RWA mounds (*cf.* Figure 2(b)) in the West Eifel (1,140 km²) and the directions of fault zones published in [19,20] (*cf.* Figure 2(c)) shows that the centers of the modes, *i.e.*, the local maxima in the histograms, denote the preferential alignment directions, while their widths indicate the corresponding directional variations. In the RWA histogram, the local maxima are separated by vertical black lines. For each mode, the average value is indicated by a solid vertical red line and the ±1 standard deviation interval surrounded by two dashed vertical red lines. More specifically, the azimuthal directions in which the RWA mound positions are aligned correspond to those of the main fault directions. The comparison of both the spatial RWA mound distribution with the significantly increased gas anomalies confirms that the RWA colony is situated in a tectonically sheared region. Although the reference site is surrounded by various forest stands, no further RWA mounds were found in those forest stands during the area-wide mapping. The statistical analyses show that the spatial distribution of RWA mounds maps tectonically active, gas-permeable strike-slip faults (*cf.* Figure 2) and that RWA mounds can be used as biological indicators of tectonically active, gas-permeable strike-slip faults [16,17,21]. This is especially useful when information about the active tectonic regime is incomplete or the resolution by technical means is insufficient [16,17]. These faults may be potential earthquake areas and are simultaneously information channels deeply reaching into the crust. Recent research shows that, among others, electromagnetic field changes emitted from the pre-focal area several days before the upcoming earthquake are discussed as one trigger mechanism for bioanomalies prior to large earthquakes [22,23,24,25]. Also mid-term to short-lived thermal infrared anomalies from the rock surface in the 8–12 μm region along thrust or strike-slip faults prior to major earthquakes are reported [26,27,28]. Consequently, an integrated approach in understanding geophysical phenomena requires consideration also of the interaction between the abiotic and biotic environment.

For a detailed and statistically well-based study, it is necessary to investigate whether changes in the ants’ standard daily routine can be correlated with local seismic events. The seismically active study area at the Neuwied Basin (Eifel, Germany) is located in an area with a complex tectonic history. Commencing during the Neogene and persisting during the Quaternary, the uplift of the Rhenohercynian Zone affects the dynamics of the study area. The currently NW-SE striking main stress direction opens pathways for geogenic gases and potential magmas [29,30,31]. Concurrently, a conjugate dextral wrench fault system (striking in 100°–110° WNW-ESE representing the extensional regime of the recent compressional stress field and a conjugated shear system trending in 140°–150° NNW-SSE) in combination with a clockwise block rotation and relatively small kinematic WSW-movements exists [29,32,33,34]. At the same time, Variscan faults as part of a conjugated shear system are reactivated [35,36]. This situation has led to a complex tectonic break clod. Due to the ongoing extension of the Cenozoic rift system in the western part of the Eurasian plate, geotectonic processes still influence the active regional tectonics and lead to a large number of low-magnitude seismic events [37,38]. In the study area, an average of about 100 earthquakes per year with magnitudes up to M 3.9 occur located on different tectonic fault regimes (strike-slip faults and/or normal or thrust faults) [29,39,40].

**Figure 3:**
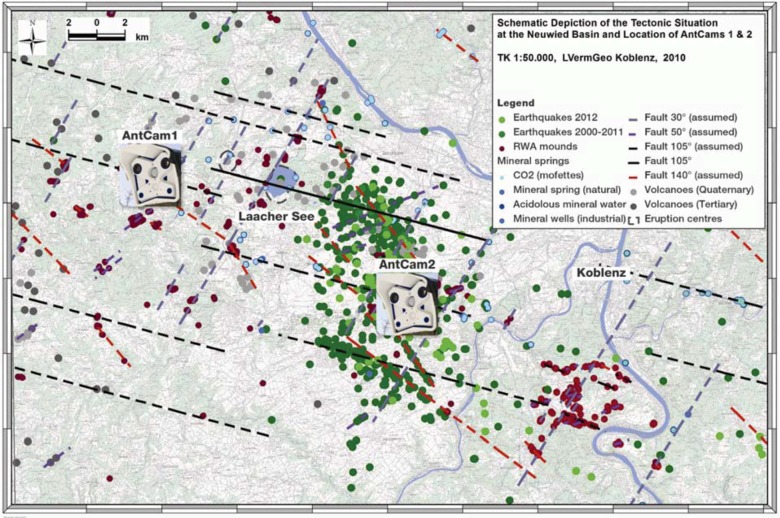
Location of both cameras (AntCam 1 and 2) within the tectonically active Neuwied basin. Shown are the East Eifel volcanic field (black dots), earthquake events (green dots) 2000–2012 [39], mineral springs and mofettes (blue dots) and a schematic depiction of the tectonically active fault systems (colored lines) [41]. Although within this region the RWA mounds have not been completely mapped so far, it is apparent that RWA mounds indicate tectonically active faults.

For three years, from 2009 to 2012, we have monitored two RWA mounds (*Formica rufa*-group; F. *pratensis* and F. *polyctena*, 24/7 by high-resolution cameras. Both mounds are located at two different fault regimes (on a WNW-ESE and on a NNW-SSE trending fault, respectively) approximately 22 km apart. The location of the two cameras is shown in Figure 3. We refer to the cameras as AntCams. During the observation period, ten magnitude > 2.0 earthquakes in a 40 km radius occurred (*cf.* Table 1).

**Table 1:** Magnitude > 2.0 earthquakes events (from 2009 to 2012) in a 40 km radius around the location of both cameras (AntCam 1 and 2) within the tectonically active Neuwied basin [39,40].

Epicenter	Date	Time (UTC)	Focal Depth (km)	Magnitude (M)	Longitude (°)	Latitude (°)	Linear distance (km) of AntCam 1 and 2 to epicenter	
							AntCam 1	AntCam 2
Bad Ems	09/10/2009	12:36:21.70	5 *	3.2	7.72	50.40	40	22
Kruft	04/11/2010	11:16:16.50	10 *	2.9	7.34	50.40	14	10
Ochtendung	08/05/2011	09:29:37.05	10 *	2.3	7.38	50.36	15	8
Plaidt	02/21/2012	02:34:57.62	11	2.5	7.38	50.38	17	6
Kobern	03/12/2012	19:00:42.66	12	2.7	7.43	50.33	22	1
Ochtendung	03/29/2012	01:23:41.06	10 *	2.2	7.41	50.34	20	2
Plaidt	05/22/2012	00:08:47.98	10 *	2.3	7.37	50.37	17	6
Lonnig	11/15/2012	16:40:24.62	10 *	2.4	7.41	50.32	21	4
Lonnig	11/22/2012	15:43:15.90	10 *	2.8	7.41	50.32	21	4
Winningen	12/16/2012	05:54:35.40	6.9	2.2	7.53	50.30	30	8

* Focal depth manually determined by [40].

## 2. Methods

### 2.1. Technical Equipment of the Monitoring Stations (AntCams)

Both AntCams are high-resolution cameras (Mobotix MX-M12D-Sec-DNight-D135N135; 1,280 × 960 pixels) equipped with both a color (day) and an infrared sensor (night). The video streams of the ants’ activities were recorded with 12 frames per second and annotated with a time stamp (UTC). The network compatible AntCams were connected to a NAS (network attached storage; 1TB) via a POE-supply (power over Ethernet). Because there is a vast amount of data (in summer up to 50 GB per day), the video streams were stored temporarily on the NAS. Every 10 to 14 days, the NAS is exchanged. Since September 2009 (AntCam 1) and January 2010 (AntCam 2), more than 45,000 hours of video streams have been recorded and stored in a video archive. 

### 2.2. Manual Analyses of Video Streams

**Figure 4:**
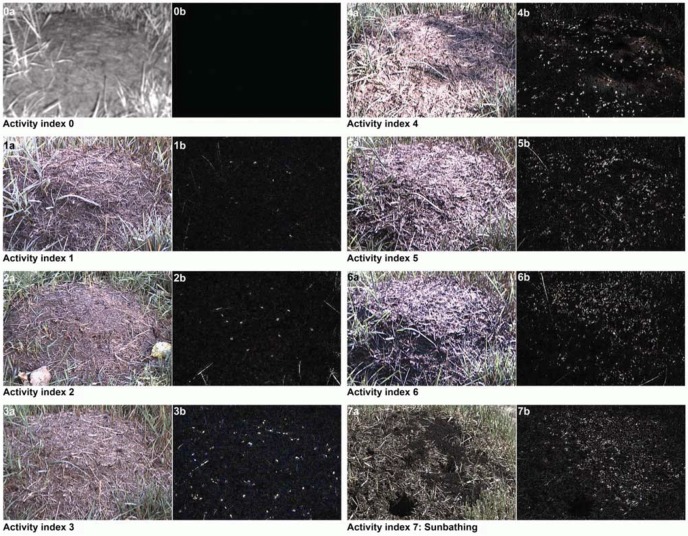
AntCam 1: Definition of activity indices with the help of difference images extracted from the master video streams to classify the ant swarm behavior on top of the mounds. Examples: Image 1a (near-infrared image acquired at night-time) shows activity index 0, meaning that there are no moving ants on top of the mound. Image 1b is the corresponding difference image between two subsequent video frames acquired at a time interval of 10 s. Because there are no moving ants on top of the mound, the difference image is black. Image 1a (color image acquired at daytime) shows activity index 1, meaning that there are only a few moving ants on top of the mound. Image 2b is the corresponding difference image. Moving ants appear as small bright dots. It is clearly apparent from the difference images that the number of RWAs on top of the mound is increasing with increasing activity index.

In the first project phase, the video streams were inspected by three independent operators who were not informed about the occurrence of earthquakes in the regarded period of time. To classify the ant swarm behavior on top of the mounds by a category *C*(*t*), eight activity categories were fixed: activity category 0 = no ants on top of the mound, activity category 6 = crowded top of the mound and activity category 7 = sunbathing (*cf.* Figure 4). The sunbathing in early spring, which takes place only for a few hours at daytime, was classified as a special activity category. The ants are very active on top of the mound within this short period, lasting approximately two weeks starting in March. Within this period of time, a large amount of the entire population is resting on top of the mound, collecting heat with their bodies and transferring the heat into the mound.

**Figure 5:**
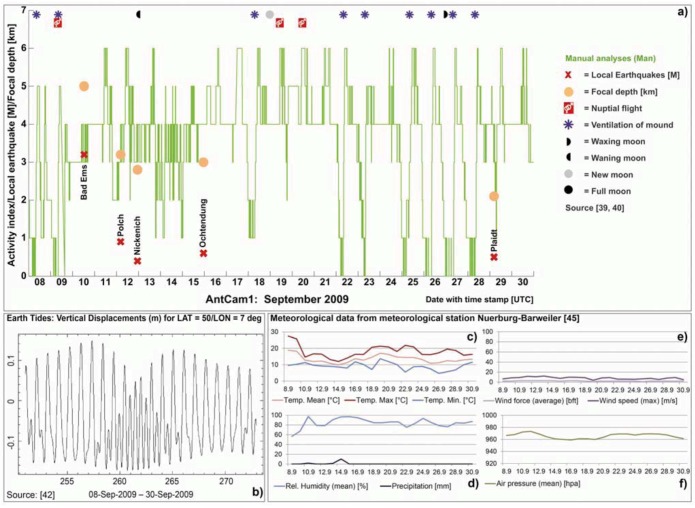
Results of manually inspected video streams (mean values generated from the three single curves) for the time segment September 08–30, 2009 (AntCam 1), in combination with local earthquakes (magnitude and depth of epicenters, *cf.* also Table 2), indicates changes in the ants’ behavior hours before the earthquake took place: e.g., the nocturnal rest phase and daily activity were suppressed September 10, 2009 and standard daily routine did not resume until the next day (*cf.* Figure 5(a) and Figure 8(b)). The spectrum of the tidal potential for the mentioned time segment is given in Figure 5(b)) and the weather data in Figure 5(c–f).

**Table 2:** Earthquake events in September 2009 in a 40 km radius of the location of AntCam 1 within the tectonically active Neuwied basin [39].

Epicenter	Date	Time (UTC)	Focal Depth (km)	Magnitude (M)	Longitude (°)	Latitude (°)	Linear distance (km) of AntCam 1 to epicenter
Bad Ems	09/10/2009	12:36:21.70	5.0 *	3.2	7.72	50.40	40
Polch	09/12/2009	04:26:57.8	3.2	0.9	7.33	50.31	17
Nickenich	09/12/2009	22:57:19.2	2.8	0.4	7.32	50.42	13
Ochtendung	09/15/2009	23:06:23.0	3.0	0.6	7.41	50.36	20
Plaidt	09/29/2009	04:38:26.4	2.1	0.5	7.38	50.38	17

* Focal depth manually determined by [39].

Every 10 minutes of each day (24 hours), a one minute sequence was evaluated at 8‐fold speed by all three operators. At this frame rate, the motion behavior of the ants is well discernible for the human eye. They scored 144 discrete numerical values as activity indices per day according to the annotated UTC time stamp. To ensure the same basis of RWA activity for the eight activity categories, the three operators were provided with master video streams showing clear examples of the eight activity categories. The results were processed in Excel, and daily activity curves (rounded mean values generated from the three single curves) connecting the discrete numerical values (activity indices) were generated. For example, if no ants were present on top of the mound, the 3 observers scored 0, showing a green curve touching the x-axis (*cf.* Figure 5(a) and Figure 8(a)). If the rounded mean value was, e.g., 4, the curve raised to the left-handed activity index 4 on the y-axis (*cf.* Figure 5(a) and Figure 8(a)). An example of the manual analysis is presented in Figure 5(a)). The Ants’ activity shows an abnormal behavior, specifically before an earthquake with a magnitude of 3.2 (Bad Ems earthquake in 40 km distance from AntCam 1 [40]). Although this magnitude can be addressed as low compared to large earthquake events, e.g., in Italy, the tectonic regime and the nature of a fault has to be taken into account. This earthquake may have triggered the tectonically active and gas-permeable “Laacher See Strike-slip Fault”, which can be tracked from the AntCam 1 position in the West, Wehrer Kessel, Laacher See, Plaidt to Bad Ems in the East [41]. In this case, bioanomalies prior to M > 2 earthquakes should be monitored in contrast to normal or reverse faults that do not show any gas-permeabilities, due to its nature. The results will be discussed in detail in Section 3.

Additionally, all abiotic (duration of sunshine, rain, snow, wind, aerating of the mound *etc.*) and biotic parameters, as the appearance and duration of predators (woodpecker, magpie, blackbird), and/or disturbing factors (boars, badger, “anting” of birds *etc.*) on top of the mound and various activities of ants (repeated nuptial flights per month, transport of larval and pupa, mound construction activities *etc.*) that might have an effect on the ants’ daily routine were read on a per-second basis and correlated with the analyzed daily activity to document the possible influence of these factors on the ants’ activity (*cf.* Figure 5(a), e.g., nuptial flights). At intervals, nest air measurements (CO_2_, helium, radon, H_2_S and CH_4_) were performed.

Earth tide data provided by the University of Bern [42] were evaluated (*cf.* Figure 5(b)). The earth’s response to the gravitational influence of both the Sun and the Moon results in two slight lunar and two solar tidal bulges (earth tides). The two bulges occur at the surface part of the earth that approximately faces the Moon and at the opposite surface part while the Earth rotates around its axis. Earth tides can be correlated with volcanic eruptions [43]. Whether earth tides also trigger earthquakes is still under discussion [44]. Another question is if earth tides might also trigger the ants’ behavior with regard to their daily routine or nuptial flights (*cf.* Figure 12(a)). 

Additionally, diurnal, monthly and mean values of a number of meteorological elements (minimum, maximum and mean temperature [°C], sunshine duration [hours], wind force [Bft] and velocity [m/s], amount of precipitation [mm] and relative humidity [%]) of the meteorological station Nuerburg-Barweiler provided by the German Meteorological Service (DWD) were considered [45]. 

Meanwhile, more than 15,000 of hours of video data of both AntCams were analyzed manually.

### 2.3. Automated Image Analysis

The key question was whether the ants’ behavioral changes and their correlation with earthquake events are statistically significant and if detection by an automated system is possible. Based on the manually gained results, an automated image evaluation routine was developed with the software Matlab and applied to the video streams to gain an objective interpretation of the ant behavior. In our image analysis system, an activity index is computed automatically in a first step based on a difference image technique. Based on a regression approach, this activity index is mapped to the range of the manually estimated activity indices to allow direct comparisons. 

The basis of the routine is a comparison of the ants’ activity on top of the mound every 10 seconds, *i.e.*, a video image is acquired every 10 seconds. In the style of the concept proposed in [46] (*cf.* [47] for further discussion of their method), for each pixel *I_uv_*(*t*) with coordinates (*u,v*) of the video image acquired at time *t*, the system determines the pixel-wise absolute difference *D_uv_*(*t*) with respect to the previous image *I_uv_*(*t ‒ ∆t*) acquired at the previous time step according to:

*D_uv_*(*t*) *= |Iuv*(*t*) *‒ I_uv_*(*t‒∆t*)|

**Figure 6:**
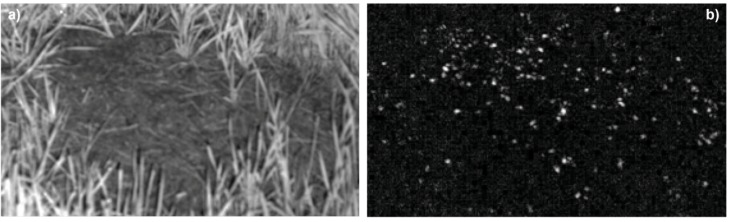
Near-infrared video image of top of the RWA mound monitored by AntCam 1 with active near-infrared illumination (**a**). Grass containing chlorophyll appears bright, as it strongly reflects near-infrared light. The ants are visible as very small dark spots. Change detection between two video frames acquired at nighttime at a time interval of 10 s (**b**). Moving ants are apparent as bright spots.

In the absolute difference image *D_uv_*(*t*), moving ants appear as bright spots (*cf.* Figure 6). The well-known disadvantage of the concept of subtracting subsequent images of a sequence, namely that objects tend to be only partially visible in the difference image [46,47], is not relevant in our scenario, due to the small size of the ants in the image of at most only a few pixels (to overcome this problem for large objects, a statistical method to generate a specific “background image” is proposed in [47]). To reduce negative influences caused by, e.g., moving blades of grass, a mask was used covering only the visible top of the mound. To compensate slight movements of the camera, e.g., due to wind, before the determination of the absolute difference image, an image registration of the current image on the previous image is performed based on normalized cross-correlation of well-distinguishable image points [48]. 

In our system, the pixel intensities of *D_uv_*(*t*), which exceed three times the pixel noise *σ*, are summed over the image region, *M*, corresponding to the top of the mound, where the ant movements are visible:

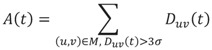


The sum value *A*(*t*) is regarded as a measure for the ants’ average activity, but it cannot be compared directly to the category *C*(*t*) defined in Section 2.2 based on manual inspection of the video sequences. To derive an activity index from the image analysis system, which is directly comparable to these categories, it has to be taken into account that images are acquired at daytime with a CMOS RGB color camera, while for images acquired at night time, a CMOS near-infrared camera sensor with active illumination is used, leading to a systematically different appearance of the images acquired at daytime and night time, respectively. Hence, the activities *A*(*t*) are subdivided into daytime values, *A_d_*(*t*), and night time values, *A_n_*(*t*). Over time intervals *T* of about 2–3 weeks duration, two sets of values:

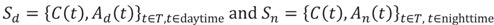

are defined. To the daytime set, *S_d_*, a first-order polynomial regression function, *p_d_*(*x*), and to the nighttime set, *S_n_*, a second-order polynomial regression function, *p_n_*(*x*), is fitted in such a way that the root-mean-squared difference between *C*(*t*) and *p_d_*(*A_d_*(*t*)) and *p_n_*(*A_n_*(*t*)), respectively, is minimized. Hence, the function values, *p_d_*(*A_d_*(*t*)) and *p_n_*(*A_n_*(*t*)), of the polynomials correspond to automatically determined activity indices, which can be directly compared to the manually inferred categories, *C*(*t*). To suppress the influence of image noise, a temporal median filter with a window length of 10 minutes is applied to the activity indices, *p_d_*(*A_d_*(*t*)) and *p_n_*(*A_n_*(*t*)).

**Figure 7:**
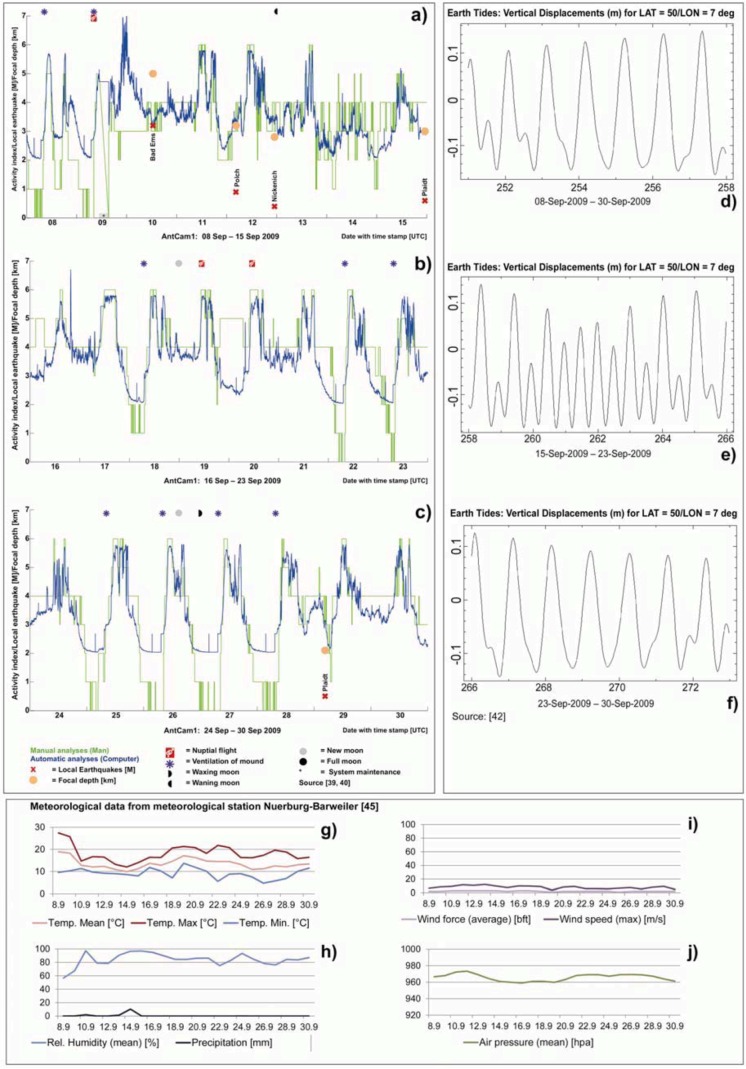
Detailed presentation of the activity index (blue: automatically determined; green: manually determined (**a**–**c**)) for the time interval September 08–30, 2009 (AntCam1) in correlation with local earthquakes (magnitude and depth of epicenters), earth tide displacement (**d**–**f**), and meteorological data (**g**–**j**).

The polynomials and the manual analysis are in good agreement (Figure 7(a–c)) and show the same abnormal behavior as the manual analysis. The result will be discussed in detail in Section 3.

**Figure 8:**
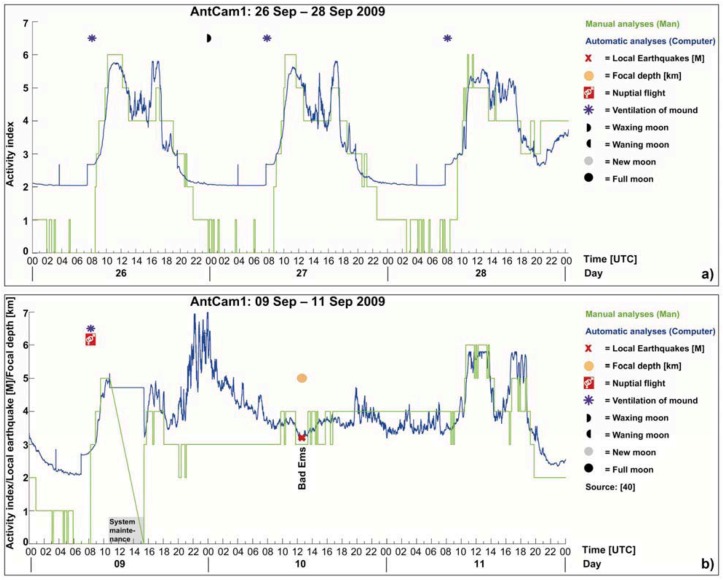
RWA have a well-identifiable standard daily routine that can be best characterized by an M-shaped curve (**a**). The correlation between the video observations and the times of occurrence of local earthquakes indicates changes in the ants’ behavior hours before the earthquake took place (**b**); the nocturnal rest phase and daily activity were suppressed and standard daily routine did not resume until the next day.

Figure 7 indicates changes in the ants’ behavior hours before the earthquake took place: the nocturnal rest phase and daily activity were suppressed on September 10, 2009, and standard daily routine did not resume until the next day (Figure 5(a–c) and Figure 8(b)). A comparison of the earth tides for the specific time interval as provided by the University of Bern (Figure 7(d–f)) and the earthquake events (Figure 7(a–c)) for the given time period suggest no possible correlation. For the given time period, ventilation of the mound (Figure 7(a–c)) takes place only at positive vertical displacements (Figure 7(d–f)). Weather conditions in September 2009 (Figure 7(g–j)) do not have a great influence on the RWA behavior. In the night from 19 to 20 September, a mismatch between the manual and the automatic analyses is apparent (Figure 7(b)). A possible explanation is the change in brightness of the material on top the mound at night. It is necessary to further analyze this issue in long-term studies.

## 3. Results and Discussion

Early results of the manually analyzed long-term observations show that ants have a well-identifiable standard daily routine that can be best characterized by an M-shaped curve: activity rises from dawn until approximately 11 A.M. Activity peaks can be observed at midday and in the late afternoon (*cf.* Figure 8(a)). During the evening, the activity is reduced again. This approach is contradictory to the observation documented so far [49]. It shows that it is possible to obtain an objective and testable null hypothesis and detailed data by a long-term, 24/7 *in situ* monitoring of the ants’ behavior using suitable technical equipment and experimental setup. A previous study [49] merely considers a serendipitous event. Additionally, no further information is provided on whether the acquired very short (30 s) single video streams have been objectively analyzed by an automated routine. 

The video observations and the times of occurrence of local earthquakes indicate changes in the ants’ behavior hours before the earthquake took place: the nocturnal rest phase and daily activity were suppressed, and standard daily routine did not resume until the next day (*cf.* Figure 8(b)). The results show that it is essential to monitor the ants’ behavior before the earthquake strikes a region. The eight minutes observation period in the run-up of the Landers M 7.4 earthquake in the Mojave Desert is too short and cannot produce statistically significant results, as it cannot be determined for such a short period of time whether the observed ants’ behavior is normal or abnormal. To define the “normal” state, it would have been necessary in that scenario to observe the ants’ behavior over longer periods of time during earthquake-free intervals in order to obtain a statistically relevant reference for observations during earthquakes. Furthermore, our observations suggest that a documentation of the aftershock activity alone is insufficient and that the conclusion that ants do not show any reaction on earthquakes, as it was made in the literature so far, is not justified based on the limited amount of data examined in previous studies [49]. Another prerequisite is to have detailed information on the active tectonic regime in the corresponding region and an understanding of geotectonic processes for interpretation of geo-biological processes, *i.e.*, RWA as bioindicators for the identification of active tectonic systems. Observations some 100 km away from the epicenter should be interpreted very carefully, since tectonic fault types and systems can change strongly within this distance. Additionally, it has not been examined so far, e.g., by geochemical analyses, if *Messor pergandei* has a close relation to tectonic fault systems. 

One of the major challenges of seismology is to determine whether fault zones generate any indication of impending earthquakes. The evolutionary mechanism of exaptation, *i.e.*, a vibration triggered early warning response or animals’ perception of these stimuli to P-waves, is reported in [5]. Precursors, as electromagnetic phenomena or the change of the gas content and concentration, might be sensed by the ants due to their electromagnetic sensitivity and ability to detect CO_2_ [16,17,18]. The objective of this methodological approach was to examine if ants show reactions prior to earthquakes. The specific mechanisms for how and why they show such reactions need to be researched in *in situ* experiments and in close cooperation with biologists.

In the night from September 19 to 20, a mismatch between the manual and the automatic analyses is apparent (Figure 7(b)). Analyses of the corresponding video material showed that the algorithm works correctly and clearly detects and counts the ants’ movements. A possible explanation is the change in brightness of the material on top of the mound at night that does not play a role for the manual analyses, but for the automated ones. It is necessary to further analyze this issue in long-term studies.

**Figure 9:**
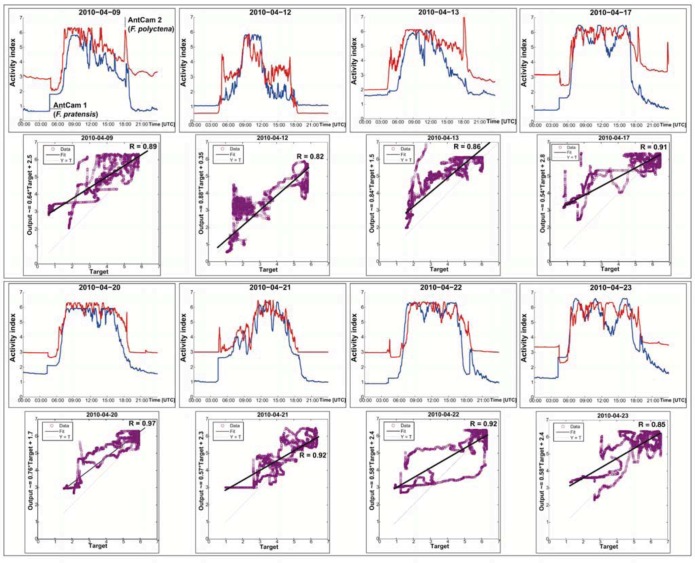
Comparison of the ants’ activity of *F. pratensis* (AntCam 1, blue curve) and *F. polyctena* (AntCam 2, red curve) at given days in April 2010 and their strong statistical correlation (R between 0.82 and 0.97).

Although we have monitored two different species (*F. pratensis* and *F. polyctena*), their daily activity patterns do not show systematic differences at days without any earthquake event and can be considered the same. According to the correlation coefficients (R between 0.82 and 0.97), the statistical correlation can be described as strong. This highly similar behavior of the two different species, as presented in Figure 9, is contradictory to previous studies and the common tenet [50,51].

External influence factors, like predators (woodpeckers, blackbirds, *etc.*) or occasionally “anting” of birds to banish parasites with formic acid, which only last for some seconds to some minutes at maximum, create only a slight increase of the peak activity, but do not have any influence on the ants’ daily routine. In practice, such influences can be avoided by fencing the mound.

**Figure 10:**
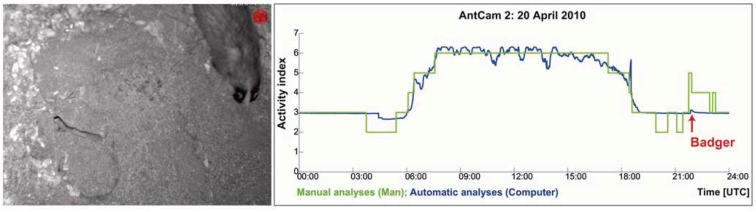
On April 20, 2010, a badger appeared on top of the mound (21:50:56 to 21:51:30 (UTC)). As can be seen, there is only a small peak activity caused by the badger, because it hits the main exit at this time interval. After this short event, the ants’ activity is calmed down to the normal activity level.

The external influence of larger animals, like badgers or boars, does not show raised activity peaks, unless a part of the mound is affected by digging (badger) or destruction (woodpecker, black bird *etc.*). Then, the ants’ activity rises to protect the mound and the population. This can be documented by a peak activity (e.g., reconstruction of the mound), which amounts to only a small fraction of the daily routine and is negligible, for the daily routines have to be considered across the whole day. Nevertheless, this extraordinary situation is documented (*cf.* Figure 10). 

During the winter season, the ants’ activity is—as it is well known—very small to non-existent. However, we have observed ants to be visible on top of the mound when an earthquake with a magnitude of about 2 occurs, despite snow cover and freezing temperatures and, moreover, with delayed activity compared to the spring-autumn seasons. The question is whether the ants’ activity will rise to a higher level for earthquakes with higher magnitudes.

**Figure 11:**
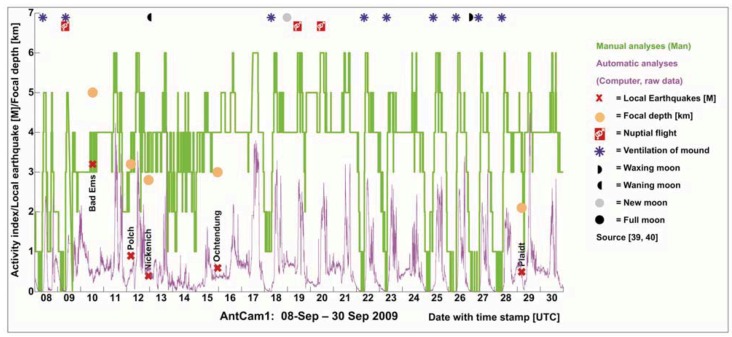
Automatically analyzed raw activity index *A*(*t*) (before polynomial regression) in comparison with the manually analyzed data. A qualitatively similar behavior is clearly apparent.

The automatically analyzed activity index (“purple curve” in Figure 11) shows that our image analysis system works independently of given manual results. Furthermore, a comparison of the manually inspected video observations (green curve) and the “raw” activity indices, *A_d_*(*t*) and *A_n_*(*t*) (prior to the mapping to the manually inferred categories *C*(*t*) based on the polynomial regression functions) (blue curve), shows that both activity curves show the same trends in the daily ants’ routine.

**Figure 12:**
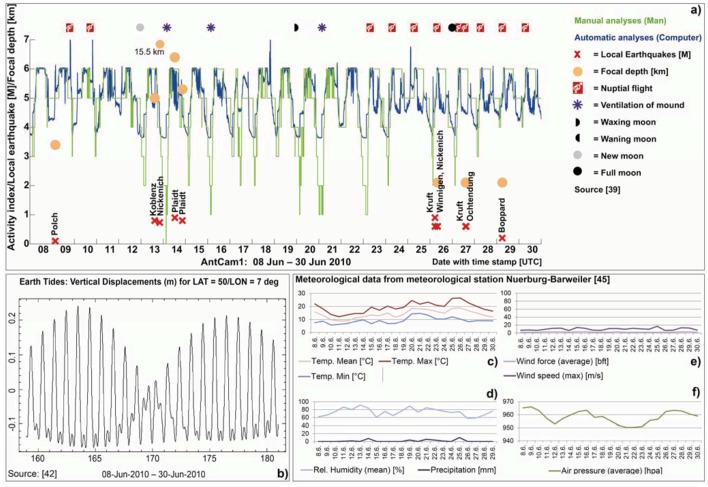
The automatically determined activity index (blue) for the time interval June 08–30, 2010 (AntCam 1) combined with the manually analyzed activities (green) in correlation with local earthquakes with a magnitude of <2. At lower magnitudes, an anomalous behavior may possibly be present as well. A comparison of the earth tides and the earthquake events suggest a possible correlation for the given time interval. An accumulation of earthquakes (June 09 (day 161); June 12–14 (days 163–165) and June 26–29 (days 177–180)) occurs only when the absolute values of the vertical displacements are at their maximum. A similar, but less pronounced, relation can be observed for nuptial flights in the given time interval (June 22–30, days 174–181). It is necessary to further analyze these relationships in long-term studies.

According to our first analyses, clearly anomalous ants behavior may occur for the local earthquake with a magnitude > 2 (Figure 8(b)). At lower magnitudes (*cf.* Table 3), an anomalous behavior may possibly be present as well (Figure 12). The question is: how RWA would react in regions with higher magnitude (M ≥ 4) earthquakes and if there is a habituation effect with regard to lower magnitudes. Possibly, the ants show reactions only for higher magnitudes. A comparison of the earth tides and the earthquake events suggest a possible correlation for the given time interval. An accumulation of earthquakes (June 09 (day 161); June 12–14 (days 163–165) and June 26–29 (days 177–180)) occurs only when vertical displacements are positive and at their maximum. Also, nuptial flights occur only when vertical displacements are positive at their maximum in the given time interval (June 22–30 (days 174–181)). It is necessary to further analyze these relationships in long-term studies.

Possibly, the ants’ behavior may also depend on the specific fault type. Strike-slip faults create deep-reaching tectonic fractures that might be gas-permeable and show mineralizations. Earthquakes occurring on these faults would trigger gas anomalies prior to the event that can propagate over a certain distance from the future quake epicenter, whereas along normal or reverse faults, due to their physical characteristics, such anomalies would be smaller. 

**Table 3:** Earthquake events in June 2010 in a 40 km radius around the location of AntCam1 within the tectonically active Neuwied basin [39].

Epicenter	Date	Time (UTC)	Focal Depth (km)	Magnitude (M)	Longitude (°)	Latitude (°)	Linear distance (km) of AntCam 1 to epicenter
Polch	06/09/2010	03:31:25.0	3.4	0.1	7.33	50.30	18
Koblenz	06/13/2010	14:46:34.7	5.0 *	0.8	7.56	50.34	30
Nickenich	06/13/2010	20:40:15.2	15.5	0.7	7.32	50.39	18
Plaidt	06/14/2010	12:32:30.9	6.4	0.9	7.34	50.40	14
Plaidt	06/14/2010	20:41:47.4	5.3	0.8	7.35	50.39	15
Kruft	06/26/2010	05:23:34.7	29.8	0.9	7.36	50.39	15
S. Nickenich	06/26/2010	05:24:18.6	15.2	0.6	7.32	50.39	13
Winningen	06/26/2010	08:15:56.3	2.1	0.6	7.51	50.32	28
N. Ochtendung	06/27/2010	14:27:27.1	2.1	0.6	7.39	50.37	18
E. Kruft	06/27/2010	15:04:37.7	2.1	0.6	7.35	50.38	15
Boppard	06/29/2010	05:59:34.0	2.1	0.2	7.62	50.23	39

* Focal depth could not be determined exactly by [39].

## 4. Conclusions

RWA bioanomalies (suppression of the nocturnal rest phase and daily activity, continuation of the standard daily routine not before the next day) have been recorded and analyzed for several earthquakes with magnitudes of up to about 3. The automated image analyses routine has been shown to provide a valuable tool to objectively identify and classify the ants’ activity on top of the mounds and an examination of its correlation with earthquakes. The results show that the automated system works and can be utilized in practice.

According to our first analyses, clearly anomalous ant behavior occurs for the local earthquake on September 10, 2009, with a magnitude of 3.2. At lower magnitudes, an anomalous behavior may possibly be present as well. The specific mechanisms for how and why they show such reactions need to be researched in *in situ* experiments and in close cooperation with biologists. An open question is whether RWA would react in the same way on earthquakes with higher magnitudes (M > 4), which have not occurred during our period of observation. This will be one topic of future research. In the next step, the analyses routine will be integrated into the AntCams to transmit the activity indices online and in real-time. After a test phase of about half of a year, we plan to install our monitoring system in tectonically more active regions.

Although the investigation and results presented here are promising, they are only a first step towards a completely new research complex. Long-term studies have to show whether confounding factors and climatic influences can clearly be distinguished. However, our early results suggest that it makes sense to consolidate and extend the research to determine a pattern for exceptional activity situations. These studies are not provided for earthquake prediction, but are an important step towards the understanding of geobiological processes.
